# Teaming Up for Asthma Control: EPR-3 Compliant School Program in Missouri Is Effective and Cost-Efficient

**DOI:** 10.5888/pcd14.170003

**Published:** 2017-05-25

**Authors:** Benjamin Francisco, Tammy Rood, Rebekah Nevel, Paul Foreman, Sherri Homan

**Affiliations:** 1 University of Missouri Health Care, School of Medicine, Child Health, Pulmonary Medicine & Allergy, Department of Child Health, Asthma Ready Communities and Teaming Up for Asthma Control, University of Missouri, Columbia, Missouri; 2 Vanderbilt University Medical Center, Pediatric Allergy, Immunology & Pulmonary Medicine, Monroe Carell Jr. Children’s Hospital, Nashville, Tennessee; 3 Missouri Department of Health and Senior Services, Missouri Asthma Prevention and Control Program and Office of Epidemiology, Jefferson City, Missouri

## Abstract

**Introduction:**

Teaming Up for Asthma Control (TUAC) is a work force development intervention to improve asthma control among children by increasing the competency of school nurses and delivering guideline-based education. We hypothesized that the knowledge and skills of participating school nurses would improve and that this change would positively affect students’ asthma health and reduce health care utilization cost.

**Methods:**

Asthma education for school nurses was provided online in a pretest/posttest format or in instructor-led groups. Students with persistent asthma were identified by using a checklist. Expert evaluators obtained student participants’ preassessments/postassessments before and after the 3 asthma checkups by the school nurse, and the assessments were compared. Health care costs were assessed using Medicaid administrative claims data.

**Results:**

A total of 54 school nurses and 178 students in Missouri participated in the TUAC evaluation from 2011 through 2014. Among school nurses who completed the online education (n = 42, 77.8%), knowledge scores significantly increased from pretest (49.1%) to posttest (90.7%, *P* < .001). Of school nurses who completed assessments on 3 children (n = 34), 91.2% met the ±6% equivalence for 1 or more assessments on forced expiratory volume in 1 second (FEV_1_) compared with the expert evaluator. At enrollment, 69.7% of students had “not well-controlled” or “very poorly controlled” asthma. Postintervention, FEV_1_ significantly improved (82.9% to 92.1% predicted), and self-reported impairment and tobacco smoke exposure significantly declined (*P* < .001). For TUAC students enrolled in Medicaid, there was an average 12-month health care cost difference (−$1,431) compared with controls.

**Conclusion:**

School nurses effectively assessed asthma status, students’ outcomes improved, and health care utilization costs declined. This evaluation contributed to program improvements to further improve health outcomes among students with asthma.

## Introduction

Pediatric asthma is a chronic inflammatory respiratory disorder that causes airflow obstruction and affects 6.3 million children in the United States (1). Children with uncontrolled asthma experience frequent exacerbations leading to a high use of acute health services, school absenteeism, and personal and societal cost. The *Guidelines for the Diagnosis and Management of Asthma* (EPR-3) provide clear recommendations for improving asthma care (2), but asthma control is often impeded because children are not receiving asthma status assessments as often as needed.

The American Academy of Pediatrics released a policy statement emphasizing the critical importance of school nurses to “identify unmet health needs of large populations of children and adolescents in the school setting” (3). Implementation of guideline-driven medical care through school settings has been effective in management of chronic diseases such as type 1 diabetes (4). Among school-aged children with asthma, school nurse screening and case management have improved school attendance (5–8), knowledge and self-management (9,10), asthma symptoms (6,11,12), and quality of life (13) and have reduced acute care utilization and costs (14). Recent studies indicated that asthma interventions in schools improved forced expiratory volume in 1 second (FEV_1_), asthma control, and use of an inhaled corticosteroid (ICS) (12,15). However, limitations included lack of resources, insufficient asthma education, competing priorities, and limited contact with primary care physicians (16–18). Distance learning technology to overcome some of these difficulties has been used successfully for school nurses’ asthma training (19) and self-care management (20,21).

Teaming Up for Asthma Control (TUAC) is a work force development intervention in Missouri aimed at promoting school nurse competency for assessing and caring for students with asthma. The TUAC intervention uses standardized tools and objective measures of airflow to provide actionable information to achieve well controlled asthma. The purpose of this evaluation was to assess the TUAC intervention on school nurses’ ability to accurately assess asthma status, improve students’ outcomes, and lower care cost. We hypothesized that the knowledge and skills of participating school nurses would improve and that this change would positively affect students’ asthma health and care utilization cost.

## Methods

The TUAC evaluation aligned intervention outputs and outcomes to EPR-3 priorities using a pretest/posttest model to assess both school nurse competency and students’ health outcomes. Before initiation, the TUAC protocol and informed consent and assent forms were approved by the Missouri Department of Health and Senior Services and the University of Missouri Hospital and Clinics Health Science institutional review boards (IRBs).

### Recruitment

School district superintendents were contacted and signed a written consent form. After obtaining superintendents’ consent, school nurses received a program overview and, if electing to participate, completed asthma training and received a step-by-step protocol and checklist for identifying students with persistent asthma (ie, high disability, emergency department visits, or hospitalization in the past 12 months; or excessive absenteeism [>5 days] due to asthma or other respiratory condition). In addition to having asthma, inclusion criteria required children and their parents or caregivers to speak, read, and understand English, the child to be developmentally and physically able to participate and have no other exclusionary respiratory condition (eg, cystic fibrosis), and children to be aged 5 to 14 years (ie, kindergarten through 6th grade). The rollout also included an update of the Missouri School Asthma Manual in 2011 (originally created in 2003) combined with an electronic letter from the state school health coordinator, introducing the opportunity to enroll children in TUAC. During 2010 and 2011, funding was received; IRB approvals and consents were obtained; and recruitment, trainings, and enrollment were conducted. The asthma assessments of students took place from April 2011 through April 2014.

After identifying students, the school nurse sent home a parent–guardian letter and information packet. The letter described an opportunity for students to enroll in the TUAC program and evaluation. Parents could opt out of having their child participate in the evaluation, the educational services, or both. Written parental consent and child assent were required to participate. For each student enrolled, the school nurse conducted a series of 3 asthma assessments at intervals of 1 to 2 weeks.

### Intervention components

The design process for the TUAC intervention consisted of 5 components. First, 4 key messages were extracted from EPR-3 recommendations as priorities for student education and self-care skills, as well as school nurse competency development: 1) use of objective measures of airflow, 2) coaching for optimal inhalation technique, 3) appropriate use of ICS, and 4) trigger reduction.

Second, a web-based training program for school nurses was produced and delivered as an alternative to instructor-led group training. The program included a mix of streaming video, interactive media, and print content. School nurses completing online asthma training completed a pretest and a posttest, and nurses completing instructor-led group training demonstrated in-person assessment of competency.

Third, school nurses were supplied with assessment tools (ie, Checklist for Identifying Persistent Asthma; selected items from the Children’s Health Survey for Asthma, Child Version [CHSA-C], with reliability estimates for activities and emotional health scales >0.70, except for children aged 8 on child activities, on which the scale was 0.68 [22]; and the Child Asthma Risk Assessment Tool [CARAT] [23], validated with low-income inner city children) and devices to measure lung function through FEV_1_, a preferred method of assessing airflow based on expert clinical guidelines, (Asma 1 digital FEV_1_/peak flow meter, Vitalograph) and inspiratory effort (In Check Dial, Clement Clark).

Fourth, each nurse received asthma care instructional media for students to use at school (16-minute video) and for students and their families to use at home (45-minute video). These programs incorporated 9 of the original 44 short (<2 minutes) animated lessons from the Interactive Multimedia Program for Asthma Control and Tracking (IMPACT Asthma-Kids), an evidence-based program cited in the EPR-3 guidelines (24). An additional video by a clinician that explained the value of school nurse asthma care was included with 4 home activity worksheets designed to complement the 4 key messages and engage parents and students to improve asthma control.

Fifth, a $20 gift card incentive was provided contingent on the parent or caregiver returning the completed worksheets to the school nurse for a discussion of ways to improve the student’s asthma. In addition, participating school nurses who completed assessments with 3 students received a $100 voucher for a school supplies company.

### Student health outcomes

Expert TUAC evaluators completed online training for the University of Missouri Hospital and Clinics Health Science Institutional Review Board, a half-day workshop in evaluation procedures and data collection, and online advanced training including video demonstration of the precise evaluation protocol. Skills were assessed at preintervention and postintervention via a comparison of measurements by an expert evaluator to those of school nurses for student FEV_1_ and inhalation technique.

At each session, the following student data were collected: self-reported ICS use and adherence, inspiratory flow rate and time compared with target time, FEV_1_% predicted (ie, FEV_1_% of the student divided by the average FEV_1_% in the population for any person of similar age, sex, race and height), and impairment (functional and sleep disruption) captured using 5 selected questions from the CHSA-C at the first and last visit. These 5 questions assessed whether asthma kept the student from the activity in the past 2 weeks and were measured on a Likert scale from 0 to 4 (0 being “not at all” to 4 being “totally”), were weighted (ie, playing × 1, running × 1, moderate activity × 2, walking × 3, and sleep disturbance × 3), and were used to create a composite functional impairment score (range, 0–40). Asthma control status was assessed by using 3 impairment measures and lung function (ie, FEV_1_) using the EPR-3 criteria as follows:

- Difficulty with moderate activity (eg, shooting hoops, bicycle riding, walking up stairs): if there was “some” limitation or more, asthma was “not well controlled.”
- Difficulty walking: if “a little bit”, asthma was “not well controlled” or “some, a lot, or totally,” then asthma was “very poorly controlled.”
- Sleep disruption: if there was “some” limitation, asthma was “not well-controlled” or if “a lot or totally,” asthma was “very poorly controlled.”
- Lung function: if FEV_1_ was 60% to 80%, asthma was “not well controlled,” or if less than 60%, it was “very poorly controlled.”

Additional assessments included 1) students’ beliefs and attitudes about asthma (eg, frustrated, sad, and embarrassed, scored from 0 to 3, ranging from 0 being “none of the time” to 3 being “most of the time”); 2) psychosocial measures (eg, stress, anger, and others don’t understand); 3) knowledge measures (scored from 1 to 5, 1 being “strongly disagree” to 5 being “strongly agree”); 4) tobacco smoke exposure (scored from 0 to 4, 0 being “not at all” exposed to 4 being “totally” exposed); environmental trigger exposures (yes/no); and 5) medication possession, documented by identification of color photographs of respiratory inhalers. Inspiratory flow and time were compared with the EPR-3 recommended flow rate of 30 liters per minute (LPM) for a calculated target time (FEV_1_ × 2 seconds) for metered dose inhalers or 60 LPM for a target time (FEV_1_ × 1 second) for dry powder inhalers. Student data were entered on Scantron forms (Scranton Corp), which were mailed to the processing center and scanned into a password-protected, secure-access Excel (Microsoft Corp) database. School nurses completed postprogram satisfaction surveys.

### Health care cost

The cost effectiveness of services provided by this program was evaluated by analyzing the state’s Medicaid administrative claims data. Total annual health care costs for children with a primary or secondary diagnosis of asthma were obtained to assess the cost trend in MO HealthNet (MHN). Next, the 12-month preintervention and postintervention costs of TUAC students continuously enrolled in MHN were compared with matched controls not participating in TUAC. The case-control match was based on age (±1.5 y), race (white, African American, or other), sex, and 12-month preintervention total health care utilization cost (within $1,000).

### Statistical analysis

The difference between preintervention and postintervention results was tested for significance. Paired *t* tests were used to compare the student health and self-management outcomes; the Wilcoxon signed-rank test was used to compare the knowledge, attitudes, beliefs, and psychosocial measures, and regression analysis was used to assess the trend in health care costs. Significance was set at an ɑ level of .05. Statistical analysis was performed using SAS statistical software (SAS Institute, Inc).

## Results

### School nurse asthma competency

**Figure 1:**
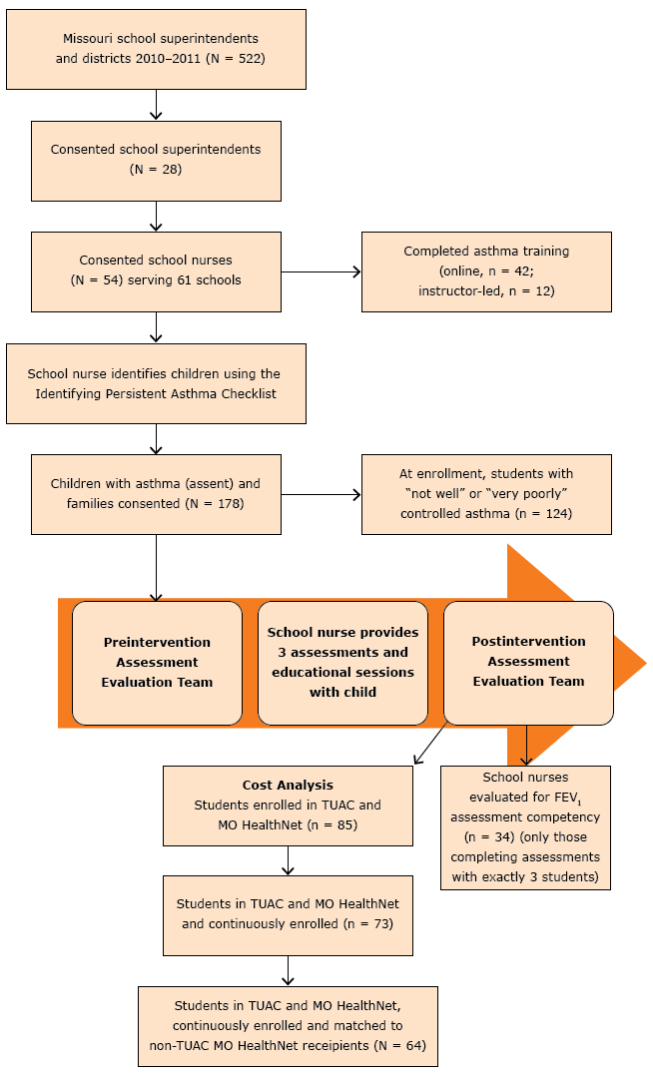
Teaming Up for Asthma Control schematic diagram, Missouri, 2011–2014.

A total of 28 school districts superintendents consented and participated in the evaluation. TUAC expert evaluators completed school nurse preintervention/postintervention skill assessments with 54 school nurses at 61 schools. A total of 178 students enrolled and received asthma assessments. The TUAC schematic is shown in Figure 1. Of the 54 school nurses, 42 (77.8%) completed the asthma education online, and knowledge scores significantly increased pretest to posttest (mean score, 49.1% vs 90.7%; *P* < .001). Overall, the school nurse group assessment of FEV_1_ was 3.5 percentage points lower than the expert group (school nurse mean = 88.4% vs expert group mean = 91.9%). Each nurse assessed from 1 to 8 students; therefore, to assess similar experience, the 34 school nurses that assessed exactly 3 students were compared with the expert evaluators; most (91.2%) were within the ±6% equivalence criteria for 1 or more of 3 FEV_1_ measurements.

### Student health outcomes

Participating students were predominately white (60.7%) or African American (33.1%), ranged in age from 6 to 14 years (mean, 9.4 y), and almost half (47.8%) were Medicaid beneficiaries (Table 1). On the basis of the initial FEV_1_ measurements or impairment scores, more than two-thirds (69.7%) of participating children met the criteria for “not well-controlled” or “very poorly controlled” asthma. Between visits 1 and 3, mean FEV_1_ for students significantly increased from 82.9% of predicted to 92.1%, an increase of 11.1% (*P* < .001), student-reported impairment scores declined 25% from 8 to 6 (*P* < .001), and tobacco smoke exposure scores declined by one-third (−33.3%) from 1.5 to 1.0 (*P* < .001) (Table 2). In addition, the FEV_1_% predicted decreased by 1.0% when the weighted functional impairment score increased by 1 showing a significant relationship between these health indicators (*P* = .009).

**Table 1:** Characteristics of Students Enrolled in the Teaming Up for Asthma Control Program, Missouri, 2011–2014

Characteristic	Number (%)
**Overall**	178 (100)
**Race/ethnicity**	
White	108 (60.7)
African American	59 (33.1)
Hispanic/other	10 (5.6)
Unknown	1 (0.6)
**Age, y**	
6–8	50 (28.1)
9–11	103 (57.9)
12–14	23 (12.9)
Unknown	2 (1.1)
**Sex**	
Male	90 (50.6)
Female	88 (49.4)
**Geographic school location**	
Urban core	122 (68.5)
Suburban	34 (19.1)
Large rural town	6 (3.4)
Small town or isolated rural area	16 (9.0)
**Health care coverage**	
Mo HealthNet	85 (47.8)

**Table 2:** Health and Self-Management Outcomes Among Students Enrolled in Teaming Up for Asthma Control, Missouri, 2011–2014

Outcomes	Overall						Paired *t*-Test				
	Pre-TUAC			Post-TUAC			Pre-TUAC, Mean (SD)	Post-TUAC, Mean (SD)	n	Difference (95% CI)	*P*
	N	Mean Value (95% CI)	SD	N	Mean Value (95% CI)	SD					
**Lung function, FEV_1_% predicted^a^**	175	78.9 (78.8–79.0)	0.453	175	88.6 (88.5–88.7)	0.457	82.9 (0.221)	92.1 (0.203)	172	9.2 (5.9 to 12.5)	<.001
**Function impaired, FI score^b^**	177	8.1 (7.1–9.2)	7.296	175	6.0 (5.0–6.9)	6.365	8.1 (7.281)	6.0 (6.379)	174	−2.1 (−3.0 to −1.2 )	<.001
**Medication, no. of ICS weekly doses^c^**	138	6.5 (5.6–7.5)	5.586	138	8.2 (7.3–9.1)	5.429	6.5 (5.586)	8.2 (5.429)	138	1.7 (0.8 to 2.6)	<.001
**Medication inhalation time minus target time, seconds**											
Inhalation time^d^	172	1.4 (1.2–1.5)	1.031	170	0.8 (0.6–0.9)	0.871	—	—	—	—	—
Metered dose inhaler (ICS)	99	1.4 (1.2–1.6)	1.038	93	0.9 (0.7–1.1)	0.808	1.4 (1.050)	0.9 (0.805)	92	−0.5 (−0.8 to −0.3)	<.001
Dry powder inhaler (ICS)	21	1.0 (0.4–1.5)	1.161	20	1.3 (1.0–1.6)	0.733	1.0 (1.170)	1.3 (0.733)	20	0.3 (−0.3 to 1.0)	.36
**Environment, tobacco smoke exposure^e^**	178	1.5 (1.3–1.7)	1.345	177	1.0 (0.9–1.2)	1.191	1.5 (1.349)	1.0 (1.191)	177	−0.5 (−0.6 to −0.3)	<.001

Abbreviations: —, not applicable (stratified by inhaler type, metered dose inhaler or dry powder inhaler [DPI]); CI, confidence interval; EPR-3, *Guidelines for the Diagnosis and Management of Asthma*; FEV_1,_ forced expiratory volume in 1 second; FI, functional impairment; ICS, inhaled corticosteroid; LPM, liters per minute; SD, standard deviation; TUAC, Teaming Up for Asthma Control.

a FEV_1_% predicted is defined as FEV_1_% of the student divided by the average FEV_1_% in the population for any person of similar age, sex, race, and height.

b FI score is a composite weighted score of activity limitations (playing, running, vigorous exercise, or walking) and sleep disruption over the past 2 weeks, ranging from 0 to 40.

c ICS weekly doses based on twice daily use for the past 2 weeks by students taking an ICS at the initial visit or who began doing so during the intervention.

d Inhalation time in seconds minus target time: difference between inhalation time and target time. Target time was calculated by using each students’ best FEV_1_ and EPR-3–recommended inspiratory flow rates for MDIs and DPIs. MDI target time: FEV_1_ (in liters) × 2 seconds (breathing in at 30 LPM, it takes 2 seconds to get a liter of air into the lungs). DPI target time: FEV_1_ (in liters) in seconds (breathing in at 60 LPM, it takes 1 second to get a liter of air into the lungs).

e Tobacco smoke exposure for the past 2 weeks based on the question, “How often do people smoke around you?” Scores ranged from 0 to 4.

Of total participating students, 120 (67.4%) reported taking ICS medication at the initial visit, and most (82.5%) used a metered dose inhaler (MDI). Inhalation effort for MDI improved for both inspiratory flow rate and time with a significant decrease in variance from optimal flow rate (*P* < .001) and for variance with optimal inspiratory flow time (*P* < .001). Inhalation effort also further improved with additional coaching. However, for students (n = 20) using a dry powder inhaler (DPI) inhalation effort did not improve significantly. The reported weekly doses of ICS significantly increased from 6.5 to 8.2 of the 14 possible doses (twice per day) among students using an MDI and who were taking ICS at the initial visit or initiated therapy during the evaluation (*P* < .001).

The student participants’ asthma attitudes and beliefs (ie, frustration, isolation, and embarrassment) significantly improved (Table 3). A small proportion of students (12.3%) reported being sad some or most of the time because of asthma and there was no significant change. Students’ psychosocial well-being significantly improved for others “understand[ing] what it is like to have asthma,” and students had less anger related to having the condition. Knowledge about which medicines to take for asthma significantly increased (*P* = .02), despite most students (85.4%) having reported at the initial assessment that they “agreed” or “strongly agreed” they knew why, how, and when to take their medication. Overall, the number of students with uncontrolled asthma decreased (−23.4%) from preintervention to postintervention from 124 (69.7%) to 95 (53.4%). Of the school nurses who participated, 87% would recommend TUAC to others.

**Table 3:** Asthma Attitudes and Beliefs, Psychosocial Measures, and Medication Knowledge Among Students (N = 178), Preintervention to Postintervention, Teaming Up for Asthma Control Program, Missouri, 2011–2014

Variable^a^		None of the Time	Little of the Time	Some of the Time	Most of the Time	Strongly Agree	*P* c
		%^b^					
**Health Attitudes and Beliefs**							
**Because of my asthma . . .**							
I am frustrated about having asthma.	Pre	37.6	24.2	20.2	18.0	—	<.001
	Post	53.9	18.5	12.9	14.6		
	Difference	16.3	−5.7	−7.3	−3.4		
I feel left out by other people.	Pre	60.1	16.3	12.9	10.7	—	.01
	Post	70.8	11.8	10.7	6.7		
	Difference	10.7	−4.5	−2.2	−4.0		
I am sad.	Pre	69.7	18.0	6.7	5.6	—	.14
	Post	77.5	10.1	6.7	5.6		
	Difference	7.8	−7.9	0	0		
I am embarrassed about having to use my inhaler.	Pre	75.8	9.6	7.3	7.3	—	.02
	Post	82.6	7.3	5.6	4.5		
	Difference	6.8	−2.3	−1.7	−2.8		
I am frustrated about having to use asthma treatments.	Pre	61.2	16.3	11.8	10.7	—	.004
	Post	73.0	10.7	7.3	9.0		
	Difference	11.8	−5.6	−4.5	−1.7		
**Psychosocial Measures**							
**How much do you agree or disagree with . . .**							
My asthma causes stress in my family.	Pre	36.5	27.0	15.7	15.7	5.1	.22
	Post	38.2	34.3	15.2	8.4	3.9	
	Difference	1.7	7.3	−0.5	−7.3	−1.2	
I am frustrated that other people don’t understand what it is like to have asthma.	Pre	17.4	24.2	12.4	32.6	13.5	.01
	Post	27.0	20.2	16.3	23.0	13.5	
	Difference	9.6	−4.0	3.9	−9.6	0	
Sometimes I get angry and ask “why is this happening to me?”	Pre	33.2	27.0	6.7	20.2	12.9	.001
	Post	42.1	36.5	4.5	10.1	6.7	
	Difference	8.9	9.5	−2.2	−10.1	−6.2	
**Medication Knowledge**							
I know which medicines to take for my asthma.	Pre	2.3	7.9	5.6	41.6	42.7	.02
	Post	2.3	2.8	6.2	37.6	51.1	
	Difference	0	−5.1	0.6	−4.0	8.4	
I know why, how, and when to take my asthma medications.	Pre	2.8	5.7	6.2	39.6	45.8	.30
	Post	3.4	5.6	5.1	40.5	45.5	
	Difference	0.6	−0.1	−1.1	0.9	−0.3	

Abbreviation: —, not applicable.

a During the past 2 weeks.

b Percentages may not sum to 100 due to rounding.

c Determined by using Wilcoxon signed-rank test.

### Health care cost

**Figure 2:**
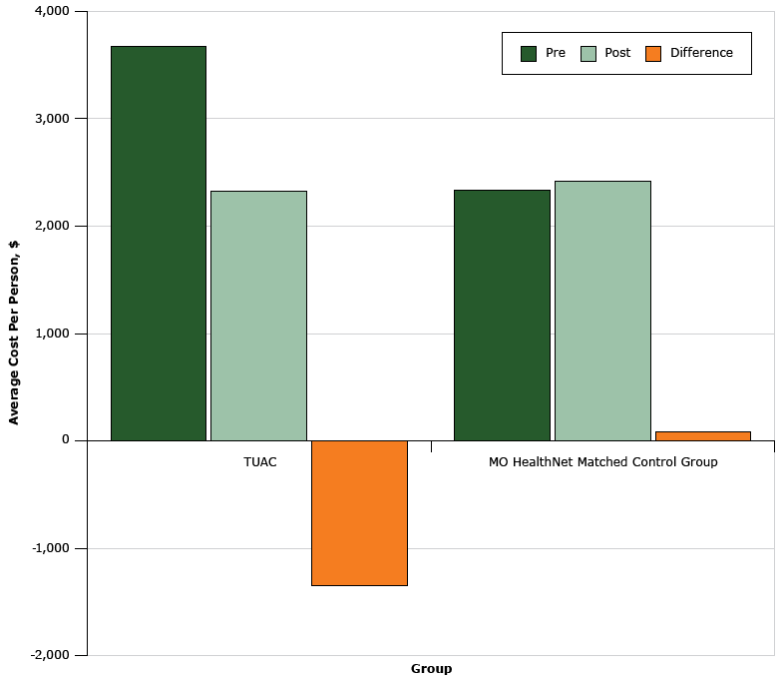
Health care utilization cost among students in the Teaming Up for Asthma Control program compared with MO HealthNet matched control group, Missouri, 2011–2014. **Table:** 

The average annual health care costs for children with a primary or secondary diagnosis of asthma enrolled in MHN showed a significant increase from 2009 to 2014 (*P* = .002). A total of 85 TUAC students were enrolled for MNH (Medicaid) services, 73 were continuously enrolled and provided 12-months of preintervention and postintervention asthma care cost information, and 64 were matched with 10,876 controls. For the TUAC students, the total Medicaid average cost declined ($1,348.48) but increased among the controls ($82.69) for an average total cost savings of $1,431.17 per TUAC student (Figure 2).

Group	Health Care Utilization, Average Cost Per Person, $		
	Preintervention	Postintervention	Difference
Teaming Up for Asthma Control	3,669.18	2,320.70	−1,348.48
MO HealthNet matched control group	2,327.47	2,410.16	82.69

## Discussion

This study demonstrates the substantial impact of educating school nurses on EPR-3 guidelines on FEV_1_, functional impairment, and quality of life. First, although other studies demonstrated application of asthma training for school nurses in single schools or small interventions (5,6,12,14), this study demonstrated a cost-effective, scalable approach to improving asthma control. Second, 7 of 10 children selected by school nurses who consented to participate had uncontrolled asthma. School nurses with EPR-3 training who used appropriate tools were successful in improving asthma control and lowering costs. Third, to our knowledge, few studies have effectively demonstrated both improvement in airflow (FEV_1_) and functional impairment, which may indicate that a weighted scale for functional impairment consistent with EPR-3 control criteria is necessary to correlate lung function and impairment. Fourth, this study demonstrated self-care education and coaching improved inhalation technique and decreased student-reported smoke exposure. Finally, few previous studies reported information on the cost of the interventions (25,26). In a separate analysis, total costs for children and youth with asthma (≤18 years of age) in MHN significantly increased from 2009 to 2014. In the study period costs among the control group increased (3.6%), whereas among TUAC students declined (−36.8%), indicating an overall 12-month average cost savings for health care utilization of $1,431 per student.

Our study’s findings are consistent with those of Mattke et al (25), who found a median self-reported ICS use of 67% compared with the TUAC students’ ICS use of 67.4%. Although the weekly doses of ICS in the homes of participating students increased significantly (*P* < .001), ICS access and adherence of participating students remained below optimal levels. Suissa et al (27) found the rate of death from asthma decreased by 21% with each additional canister of ICS used in the previous year (adjusted rate ratio, 0.79; 95% confidence interval, 0.65–0.97).

This study has limitations. Student-reported measures are subject to self-report measurement bias, and not all children and their families invited subsequently consented to participate; therefore, a nonresponse bias could exist if the children and their families who did not consent differed significantly from participants. The number of students assessed by the nurses varied, so the experience and skills of the school nurses also may have varied. Nevertheless, of the school nurses who enrolled exactly 3 students, most of the FEV_1_ measurements met the equivalence to expert criteria. Although the weekly doses of ICS in the homes of participating students increased significantly (*P* < .001), ICS access (76%) and adherence of participating students remained below optimal levels, demonstrating an area for improvement. Lastly, both school nurses and students and their families received incentives to participate. Fewer school nurses and students may have participated without the incentives; however, because caring for children with asthma is part of the school nurse’s role and two-thirds of the students had uncontrolled asthma, most may have considered participating without the incentives.

A substantial time lapse between student assessment and data analysis was observed during the first phase of the evaluation. Such a delay impeded rapid response to intervene with students who were experiencing a high level of impairment and risk. In response, the project team began building a secure web-based application to collect, rapidly transfer, and analyze health assessment data from school nurses and link them to health care centers. A formative evaluation associated with building the web-based application indicated that the term “asthma assessment” lacked meaning to most family members; TUAC incorporated this feedback and has since revised its terminology to “asthma check-up” as a more acceptable and nonthreatening term for school nurses, parents, students, and health care providers.

School nurse–delivered asthma education can improve student health outcomes and medical utilization, but outcomes vary widely. Interventions integrating guideline-based education and assessments are limited, and little is known of the effects on lung function, asthma control, and psychosocial outcomes. School nurses demonstrated significant knowledge gains in asthma assessment through online education and expert mentoring and identified a substantial number of children with persistent asthma. Through school nurse–delivered “checkups,” lung function by FEV_1_, impairment, and psychosocial indicators significantly improved for the students.

Overwhelmingly, nurses who participated in our study said they would recommend TUAC to others (87%), and most clinical outcomes were favorable and significant. We propose this innovative, cost-efficient approach for substantially increasing the frequency of asthma assessments and education for self-care by school nurses. If coupled with a strategy for collaboration with medical home providers and specialist clinical teams, this approach will lead to earlier recognition of asthma-related risk and impairment, increased use and effectiveness of ICS monotherapy, improved inhalation technique, and improved asthma control. This approach has reduced impairment and increased FEV_1_ at considerably lower costs and will likely improve school attendance and achievement of those children receiving EPR-3–compliant care.
